# Effects of endophytic fungi on parasitic process of *Taxillus chinensis*

**DOI:** 10.1038/s41598-022-11940-z

**Published:** 2022-05-11

**Authors:** Lisha Song, Limei Pan, Ni Jiang, Jine Fu, Lingyun Wan, Shugen Wei

**Affiliations:** Guangxi Botanical Garden of Medicinal Plants, Nanning, 530023 China

**Keywords:** Microbiology, Systems biology

## Abstract

*Taxillus chinensis* (DC.) Danser is an extensively used medicinal shrub in the traditional as well as modern systems of medicines. It is a perennial hemiparasitic plant, which is difficult to propagate artificially because of its low parasitic rate. Successful parasitism of parasitic plants is to fuse their tissues and connect their vasculature to the host vasculature building a physiological bridge, which can efficiently withdraw water, sugars and nutrients from their host plants. It is reported that endophytic fungi play an important role in cell wall degradation and fusion, which is the key forming process of the physiological bridge. Therefore, in this study, the endophytic fungi from *T. chinensis* of different hosts were isolated, and then the organisms that could degrade the main components of the cell walls were screened out using a medium consisting of guaihuol and cellulose degradation capacity. The results showed that five strains were screened out from 72 endophytic fungi of *T. chinensis* which with high enzyme activities for lignocellulosic degradation. The laccase and cellulase activities of five strains reached their peaks at day 7, and the highest enzyme activities of these two enzymes were found in strain P6, which was 117.66 and 1.66 U/mL, respectively. Manganese peroxidase of strain 4 and lignin peroxidase of strain N6 also reached their peaks at day 7 and were the highest among the 5 strains, with enzyme activities of 11.61 and 6.64 U/mL, respectively. Strains 4, 15, 31, N6 and P6 were identified as *Colletotrichum* sp., *Nigerrospora sphaerica*, *Exserohilum* sp., *Diaporthe phaseolorum* and *Pestalotiopsis* sp., respectively, according to their morphological and molecular biology properties. The endophytic fungi may secrete efficient cell wall degradation enzymes, which promote the dissolution and relaxation of the cell wall between *T. chinensis* and host, thus contributing to the parasitism of *T. chinensis*.

## Introduction

*Taxillus chinensis* (DC.) Danser belonging to the Loranthaceae family is mainly distributed in the southern and southwestern areas of China. The dry stems and branches with leaves of *T. chinensis* are commonly used ingredients in traditional Chinese medicinal and are called “Sang Ji Sheng” in China. *T. chinensis* has a high medicinal value. It is used for relief from rheumatic conditions, reinforcement of the liver and kidneys, strengthening of tendons and bones and prevention of abortions^1^. *T. chinensis* is hemiparasitic plant with diverse hosts^2^. Meanwhile, *T. chinensis* is also used as raw materials for making parasitism tea in China, which is a traditional Chinese food healthcare tea and it is exported to nearly 30 countries in Southeast Asia^3^. Therefore, the demand of *T. chinensis* is constantly rising in the global herbal market due to its immense therapeutic potential. However, *T. chinensis* is mainly derived from wild resources, which cannot fully meet the increasing demand of the market. The artificial cultivation of *T. chinensis* is an effective measure to balance market supply and demand.

However, *T. chinensis* is a perennial hemiparasitic plant, which is difficult to propagate artificially because of its low parasitic rate. An essential component of parasitic success in parasitic plants is the ability to fuse their host cell walls and connect their vasculature by a specialized organ known as the haustorium, thus forming a physiological bridge^4–7^. This allows transfer of not only water and nutrients into the parasite but also macromolecules, including mRNAs^8^ and proteins^9^. Thus, the host’s cell wall is the first barrier to the formation of a physiological bridge. Interestingly, studies have found that penetration of the haustorium does not cause significant damage to host plant cells. For example, the haustorium of *Striga hermonthica* does not cause endodermis cell damage of the host plant during penetration^10–12^. This can be achieved in different ways. In most parasitic plants of the Orobanchaceae family, a large number of enzymes related to cell wall degradation have been found in the parasitic process^12,13^. For example, pectin methyl esterase which can degrade pectin, is found at the hastorium puncture sites of *Oroanche cumana* Wallr. and *Phelipanche aegyptiaca* Pers.^14^. Other cell wall modifiers, such as dilatases and enzymes with transglucanase activity have been shown to peak during the penetration period of dodder infection of WM-xylglucan. In contrast, inhibition of these WM-xylglucan modifiers reduced the chance of successful dodder invasion^15^. Therefore, enzymes related to cell wall degradation play an important role during the parasitic process. However, the origin of these enzymes has not been further explored.

*T. chinensis* is a hemiparasitic plant with diverse hosts^2^, our previous works found that *T. chinensis* from different hosts had rich endophytic fungi resources. It has reported that endophytic fungi plays an important role in host plant infection and colonization strategies. Many studies also have shown that endophytic fungi has a strong ability to produce enzymes related to cell wall degradation^16,17^. For example, the endophytic fungi that have been reported to be xylanase producers include *Alternaria alternata*^18^, *Hymenoscyphus ericae*^19^, and *Aspergillus terreus*^20^. De Almeida et al.^21^ selected strains from the Acremonium endophyte species for hemicellulases and cellulases production. Suto et al.^22^ isolated 155 strains of fungi that produced xylanases. Harnpicharnchai et al.^23^ purified a thermotolerant β-glucosidase from an endophytic *Periconia* sp. from 14 plant species. Robl et al.^16^ isolated 110 strains endophytic fungi that produced hemicellulases and related enzymes. Other studies have involved the selection of new isolates using extracellular enzymes as selection parameters for plant growth promotion. Such as, Silva et al.^24^ investigated fungi isolated from *Annona* spp., while Luz et al.^25^ employed isolates from *Passiflora edulis*. Queiroz et al.^26^ study found that regarding the CAZymes in the secretomes of the analyzed fungi, the most abundant CAZym families were glycosyl hydrolase and serine proteases. And this study demonstrates that secretomes of endophytic and nonendophytic fungi share an arsenal of proteins important in the process of infection and colonization of host plants. In addition, Jaklitsch et al.^27^ performed a phylogenetic analysis of the plant cell wall-degrading carbohydrate-active enzymes and auxiliary proteins encoded in the genomes of nine species of *Trichoderma* that are members of three major infrageneric cladesplus twelve other Hypocreales fungi. Druzhinina et al.^28^ have investigated the evolution of proteins required for plant cell wall degradation in nine *Trichoderma* genomes and found an unprecedented number of lateral gene transfer (LGT) events for genes encoding these enzymes.

It is well known that the main components of plant cell walls are lignin and cellulose. The role of fungi in degrading these substances has been widely reported, but these reports have been mainly focused on some types of wood rotting fungi, such as the white rot fungus *Phanerochaete chrysosporium* Burds, *Ceriporiopsis subvermispora* (Pila T) Gilb. & Ryvarden, and the brown rot fungus *Postia placenta* (Fr.) M.J. Larsen & Lombard, etc^29–31^. White rot fungi are considered to be the most effective and are the main microorganisms for lignin degradation. White rot fungi have formed a unique degradation system in the long-term biological evolution process. Laccase, manganese peroxidase and lignin peroxidase jointly constitute the lignin degradation enzyme system of white rot fungi^32^, and it can degrade all the components of plant cell walls, including lignin, cellulose and hemicelluloses^33,34^.

Currently, little is known about the mechanism of endophytic fungi in plant cell wall degradation, but the role of wood rot fungi in lignin degradation provides a reference for studying the parasitic mechanism of *T. chinens*is from the perspective of endophytic fungi. This method showed that the white rot fungi strains had the highest ability for lignin and cellulose degradation^35–37^. In the present study, we isolated endophytic fungi from haustorial roots of different hosts, these strains were selected for qualitative determination of enzyme activities of laccase, lignin peroxidase, manganese peroxidase and cellulase. Exploring the mechanism of the endophytic fungi of *T. chinensis* in the cell wall degradation during the parasitic process not only provides practical guidance and theoretical basis for the propagating of *T. chinensis*, but it can also provide new research ideas for the parasitic mechanisms of other parasitic plants, such as *Cuscuta chinensis*, *Striga asiatica*, *Cistanche deserticola*.

## Results

### Isolation of endophytic fungi

A total of 147 strains were isolated from *T. chinensis* (DC.) Danser composed of seven host species. These were 32 strains of *M*orus *alba* L., 17 strains of *P*runus *salicina* Lindl., 12 strains of *Diospyros kaki* Thunb., 26 strains of *Dimocarpus longan* Lour., 10 strains of *Phellodendron chinense* Schneid., 27 strains of *Dalbergia odorifera* T.Chen and 23 strains of *B*auhinia *purpurea* Linn.. A total of 72 strains of strains with different morphological types were selected and the activities of lignin degrading enzyme and cellulase were assessed by using the plates.

### Determination of lignin degrading enzyme activity by plate

**Figure 1:**
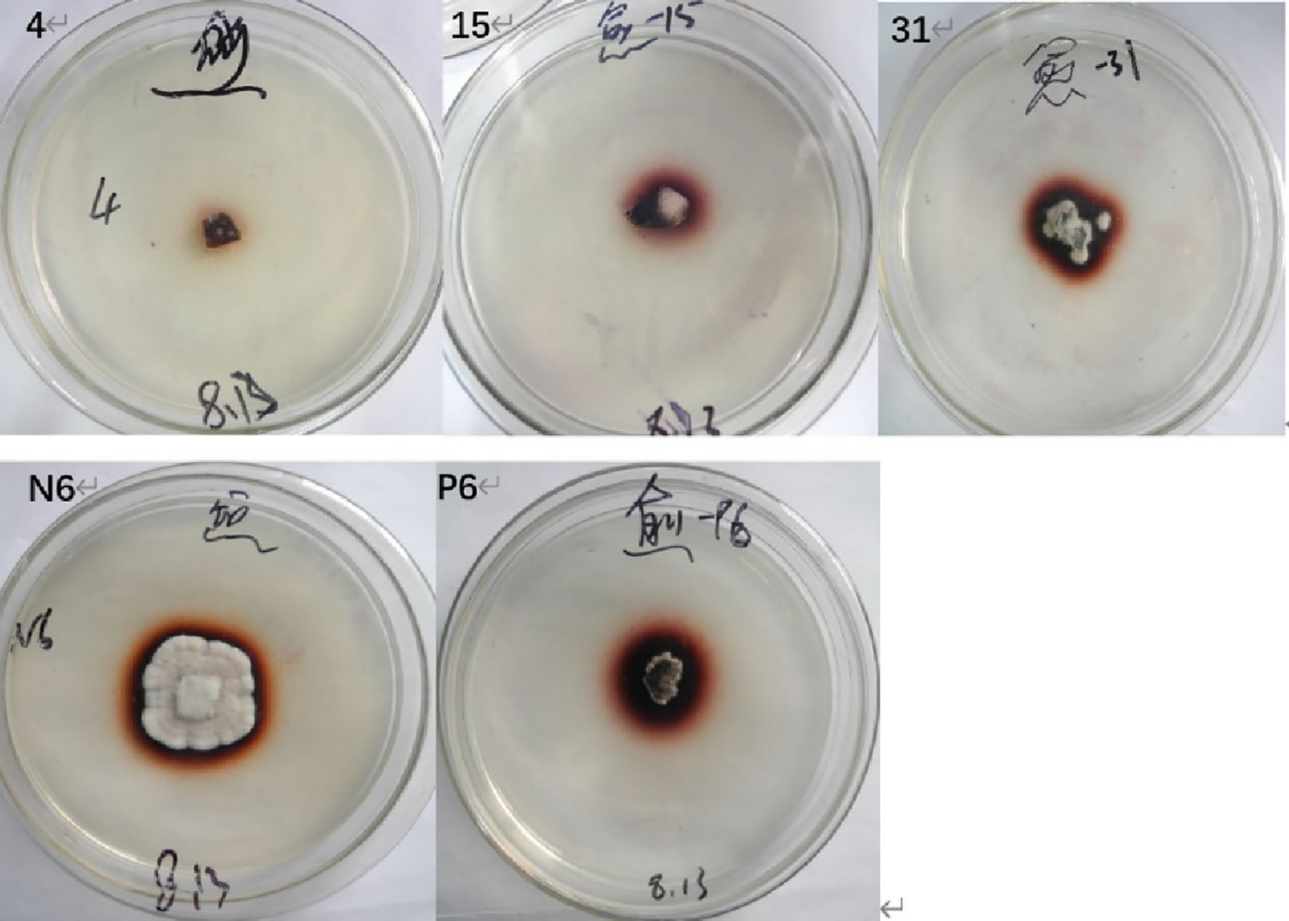
Growth diagrams of different strains on the PDA color plate on the 7th day. *Note*: 4 represents *Colletotrichum acutatum*; 15 represents *Nigrospora sphaerica*; 31 represents *Exserohilum rostratum*; N6 represents *Diaporthe phaseolorum*; P6 represents *Pestalotiopsis arceuthobii*.

The microorganisms producing chromogenic circle on the selective medium with guaiacol as indicator had the ability to degrade lignin, and the hyphae of laccase producing strains growing on the medium produced an obvious reddish brown coloration. 72 strains were cultured on guaiacol selective medium plates for 11 days, and the discoloration of each plate was observed and recorded on days 3, 5, 7, 9 and 11. The results showed that 11 strains had neither colony circle nor chromogenic circles. 17 strains had the ratio of colony circle diameter to chromogenic circle diameter of less than 1. There were 26 strains with the ratio of colony diameter to chromograph diameter greater than 1. There were 7 strains with chromogenic circles but without colony circles. There were 11 strains with colonies but without chromogenic circles. According to reference^38^, research shows that the ratio of the diameter of colony circle to chromophore can be used to judge whether the bacteria can selectively degrade lignin. If the ratio is less than 1, the bacteria can selectively degrade lignin. In summary, after qualitative preliminary screening and re-screening by the guaiacol method, a total of 5 strains with large and obvious chromogenic circles were screened out from 24 fungal strains (fungal strains with a ratio of colony circle diameter to chromogenic circle diameter of less than 1 and strains with chromogenic circles but without colonies) for subsequent enzyme activity tests (Table 1). It can be seen from Table 1 that the colonies and chromogenic circles of these 5 plants also increased with the growth time period, and neither of them increased until day 11. Figure 1 shows the growth of the five strains on the PDA color plate after day 7.

**Table 1:** Diameter changes of colony and chromogenic circles of different strains on selective media indicating lignin degradation ability over time (cm).

Strains	3 d		5 d		7 d		9 d		11 d	
	C	CR	C	CR	C	CR	C	CR	C	CR
4	0.00 ± 0.00a	1.30 ± 0.17a	0.00 ± 0.00a	1.50 ± 0.06a	0.00 ± 0.00a	1.70 ± 0.06a	1.70 ± 0.06a	2.90 ± 0.11a	1.70 ± 0.06a	2.90 ± 0.11a
15	0.00 ± 0.00a	1.30 ± 0.06a	0.00 ± 0.00a	1.70 ± 0.12ab	0.00 ± 0.00a	2.20 ± 0.05b	2.20 ± 0.10b	2.80 ± 0.11ab	2.20 ± 0.10b	2.80 ± 0.11ab
31	1.30 ± 0.14b	2.10 ± 0.12b	1.50 ± 0.06b	2.80 ± 0.23c	1.70 ± 0.12b	3.00 ± 0.06c	2.60 ± 0.12c	3.10 ± 0.06ac	2.60 ± 0.12c	3.10 ± 0.06ac
N6	1.00 ± 0.05c	0.00 ± 0.00c	1.50 ± 0.17b	2.10 ± 0.12bd	2.30 ± 0.06c	3.50 ± 0.05d	3.10 ± 0.12d	4.50 ± 0.17d	3.10 ± 0.12d	4.50 ± 0.17d
P6	0.00 ± 0.00a	1.80 ± 0.12bd	0.00 ± 0.00a	2.20 ± 0.23be	0.00 ± 0.00a	3.00 ± 0.12ce	1.50 ± 0.17ae	3.40 ± 0.12ce	1.50 ± 0.17ae	3.40 ± 0.12ce

C is for Circle of the colony; CR is for Color ring. Mean values are marked with different lower case letters are significantly different (*P* < 0.05).

### Cellulose degrading enzyme activity assay

**Figure 2:**
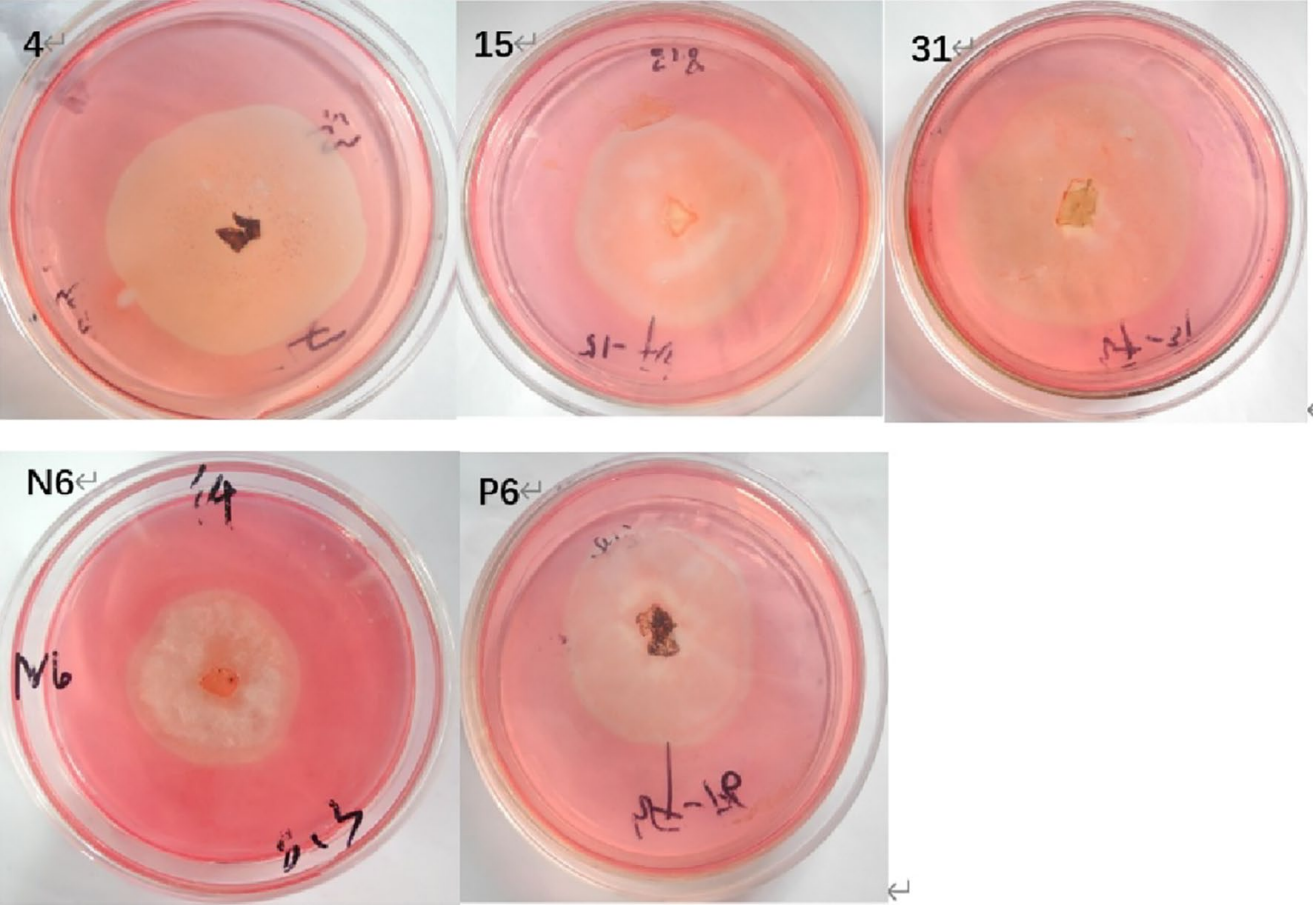
Growth diagrams of different strains on the Congo red agar plates on the day 7. *Note*: 4, 15, 31, N6 and P6 represent *Colletotrichum acutatum, Nigrospora sphaerica*, *Exserohilum rostratum*, *Diaporthe phaseolorum*, and *Pestalotiopsis arceuthobii*, respectively.

With respect to the cellulose solid medium plate culture from 72 strains at day 11, inspection of the plates on days 3, 5, 7, 9 and 11, showed 14 strains had no transparent colony circles. The ratio of colony diameter to transparent circle diameter was less than 1 in 58 strains. The other strains had the ratio of colony free diameter to transparent diameter of greater than 1. Similarly, strains with the same lignin degrading enzyme were selected from strains with the ratio of colony circle diameter to transparent circle diameter less than 1 for subsequent enzyme activity test (Table 2). As can be seen from Table 2, these 5 strains gradually grew larger with time, and remained unchanged until 9 days while maintaining the original value range. Figure 2 shows the chromogenic growth of these five strains on cellulose solid medium at day 7.

**Table 2:** Diameter changes of colony and transparent circles of different strains on selective media indicating cellulose degradation ability over time (cm).

Strains	3 d		5 d		7 d		9 d		11 d	
	C	T	C	T	C	T	C	T	C	T
4	4.50 ± 0.06a	5.00 ± 0.06a	5.00 ± 0.06a	5.00 ± 0.06a	5.20 ± 0.17a	5.20 ± 0.17a	5.20 ± 0.17a	5.20 ± 0.17a	5.20 ± 0.17a	5.20 ± 0.17a
15	3.90 ± 0.05b	4.60 ± 0.06b	3.90 ± 0.23b	4.60 ± 0.06b	4.20 ± 0.17b	4.60 ± 0.06b	4.20 ± 0.17b	4.60 ± 0.06b	4.20 ± 0.17b	4.60 ± 0.06b
31	4.60 ± 0.06ac	5.70 ± 0.23c	5.70 ± 0.17c	5.70 ± 0.23c	5.80 ± 0.29c	6.00 ± 0.06c	5.80 ± 0.29c	6.00 ± 0.06c	5.80 ± 0.29c	6.00 ± 0.06c
N6	3.70 ± 0.05bd	3.90 ± 0.06d	3.90 ± 0.06bd	4.20 ± 0.06d	3.90 ± 0.18bd	4.20 ± 0.17d	3.90 ± 0.18bd	4.20 ± 0.17d	3.90 ± 0.18bd	4.20 ± 0.17d
P6	4.40 ± 0.12ae	4.60 ± 0.12be	4.60 ± 0.12ae	4.80 ± 0.12ab	4.60 ± 0.00be	4.80 ± 0.12be	4.60 ± 0.00be	4.80 ± 0.12be	4.60 ± 0.00be	4.80 ± 0.12be

C is for Circle of the colony; T is for transparent circle. Mean values are marked with different lower case letters are significantly different (*P* < 0.05).

### Identification of strains

**Figure 3:**
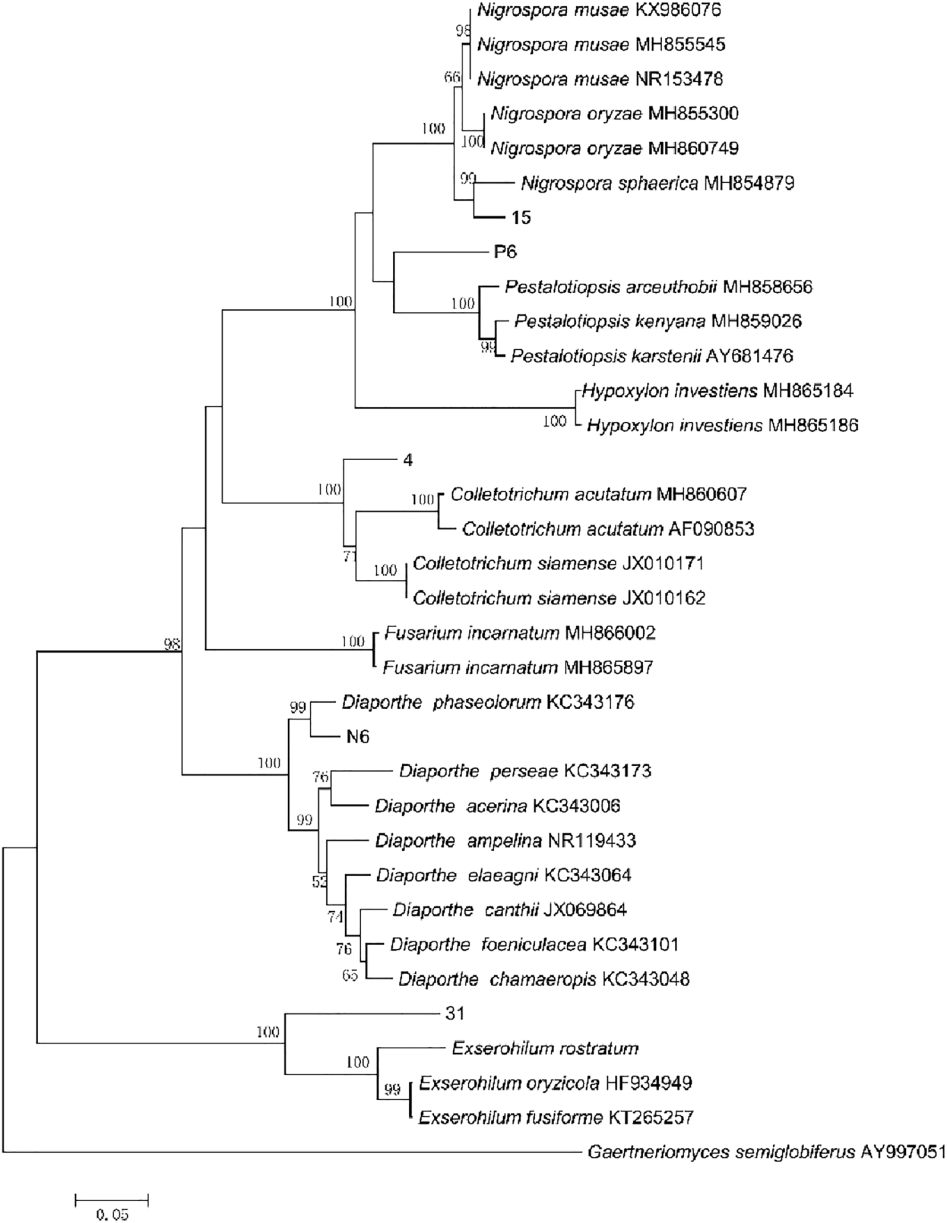
Phylogenetic tree constructed based on ITS rDNA and beta-tubulin sequences. Bootstrap values > 50% (1000 replication) are given at the nodes. *Gaertneriomyces semiglobiferus* was used as the outgroup.

A phylogenetic tree was constructed by combining ITS rDNA sequences and beta-tubulin sequences, and the results (Fig. 3) showed that stains 4, 15, 31, N6 and P6 were correlated with *Colletotrichum* sp., *Nigrospora sphaerica* (CBS MH854879), *Exserohilum* sp., *Diaporthe phaseolorum* (CBS KC343176), and *Pestalotiopsis* sp.. These species in the same branch and the similarity is 99–100%. Therefore, these five strains were identified as *Colletotrichum* sp., *N. sphaerica*, *Exserohilum* sp., *D. phaseolorum* and *Pestalotiopsis* sp., respectively. A phylogenetic tree of five isolates from this study and sequences from GenBank using combined ITS, TUB2 genes with Neighbor-Joining method analysis is shown in Fig. 3. The percentage of replicate trees in which the associated taxa clustered together in the bootstrap test (1000 replicates) is shown above the branches.The tree is drawn to scale, with branch lengths in the same units as those of the evolutionary distances used to infer the phylogenetic tree. The evolutionary distances were computed using the p-distance method and are in the units of the number of base differences persite. All taxa were distributed in five clusters, namely phylum Ascomycota within five orders, five genus. Strain P6 composed a supported clade, which closed with strain 15. Strains N6 and 4 composed a supported clade (98% bootstrap support), which closed with strains P6 and 15. Sequences of these five strains were submitted to the GenBank database and the accession numbers obtained (ITS and beta-tubulin) are MZ2823601/MZ964759, MZ2823600/MZ934421, MZ2823597/MZ934418, MZ2823599/MZ934420 and MZ2823598/MZ934419, respectively.

### Activity analysis of lignin degradation enzyme system

#### Laccase activity

**Figure 4:**
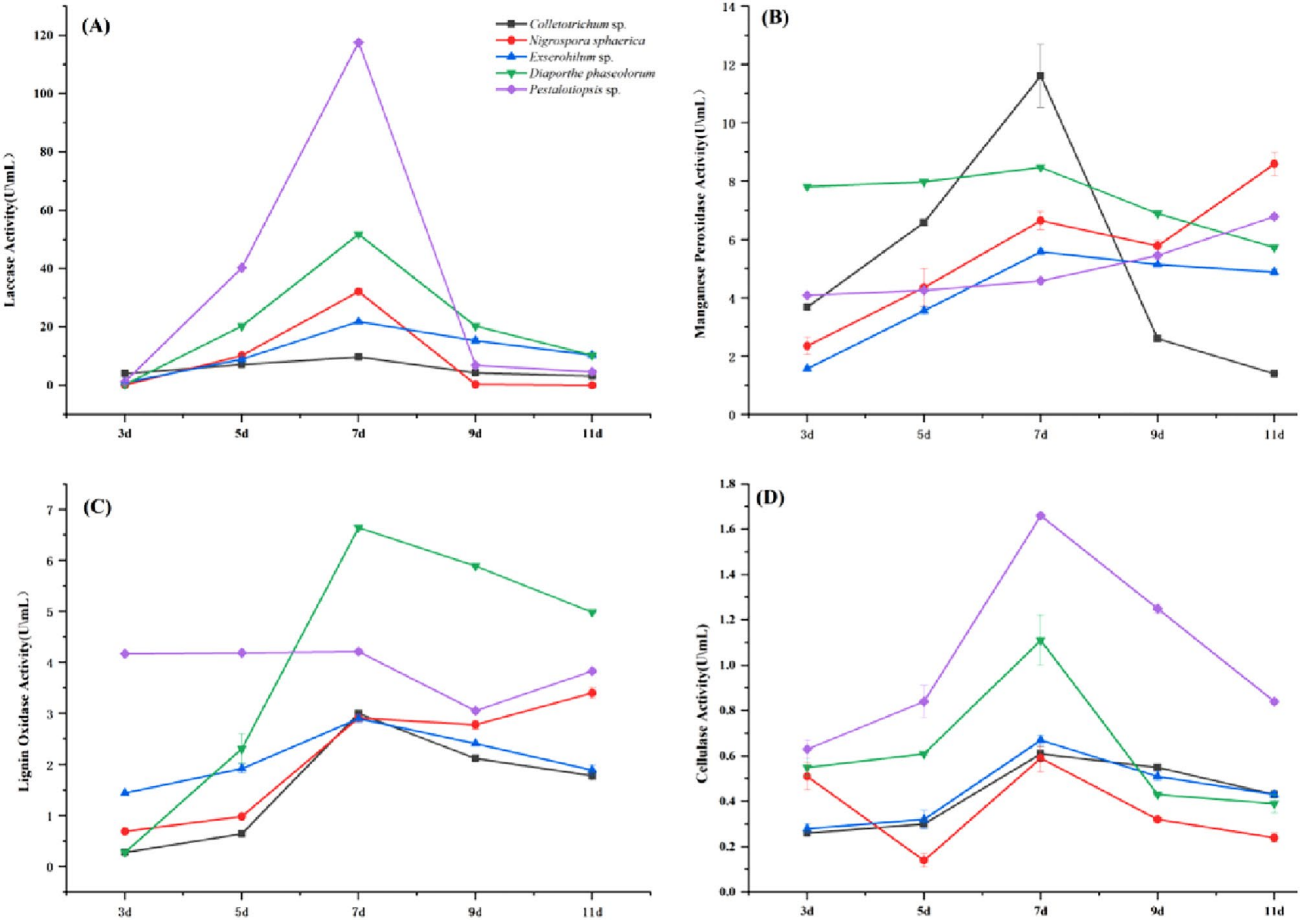
Variations of relevant enzyme activities produced by different endophytic fungi over time during lignin and cellulose degradation: (**A**) laccase activity; (**B**) manganese peroxidase activity; (**C**) lignin oxidase activity; (**D**) cellulase activity.

As can be seen from Fig. 4A, the peak time of laccase production of these five strains was on day 7, and this began to decline from day 9. The laccase production capacity of the 5 strains was significant (*P* < 0.05). Among them, *Pestalotiopsis* sp. had the strongest laccase production capacity (117.66 U/mL), followed by *D. phaseolorum*, *N. sphaerica* and *Exserohilum* sp. (51.82, 32.11 and 21.85 U/mL, respectively), and *Colletotrichum* sp. had the weakest laccase production activity (9.76 U/mL). The order of enzyme activities of the five strains was *Pestalotiopsis* sp. > *D. phaseolorum* > *N. sphaerica* > *Exserohilum* sp. > *Colletotrichum* sp.

#### Manganese peroxidase activity

As shown in Fig. 4B, the highest value of manganese peroxidase production by *Colletotrichum* sp. was 7 days, with the enzyme activity of 11.61 U/mL, followed by *D. phaseolorum* and *Exserohilum* sp., with the highest enzyme activities of 8.47 and 5.58 U/mL, respectively. On the other hand, *N. sphaerica* and *Pestalotiopsis* sp. had the highest enzyme production capacity at day 11, and the enzyme activities were 8.59 and 6.79 U/mL, respectively.

#### Lignin oxidase activity

As shown in Fig. 4C, the lignin peroxidase activity curves of *D. phaseolorum*, *Colletotrichum* sp., *Exserohilum* sp. all increased first with the change of time, reached the peak of enzyme activity and then began to decline. The highest lignin peroxidase production capacity of these three strains was at 7 days, and the enzyme activities were 6.64, 3.0 and 2.9 U/mL, respectively. The lignin peroxidase activities of *Pestalotiopsis* sp. and *N. sphaerica* increased at first, then decreased and then began to increase again on day 11, with the highest enzyme activities of 4.21 and 3.4 U/mL, respectively. The order of lignin peroxidase activity of the five strains was *D. phaseolorum* > *Pestalotiopsis* sp. > *N. sphaerica* > *Colletotrichum* sp. > *Exserohilum* sp., and the values of the enzyme activities at the lowest and the highest values of the five strains were significantly different (*P* < 0. 05).

#### Cellulolytic enzymes

From Fig. 4D, it can be seen that the cellulose enzyme activities of the four strains, *Pestalotiopsis* sp., *D. phaseolorum*, *Exserohilum* sp. and *Colletotrichum* sp. first rise until a peak is reached at day 5, and then decline. However, for *N. sphaerica* a sudden drop is seen at day 5 and then a rise at day 7 followed by a decrease. In all the five strains, cellulase production at day 7 was at its highest. The cellulase activites were 1.66, 1.11, 0.67, 0.61 and 0.59 U/mL at day 7 in *Pestalotiopsis* sp., *D. phaseolorum*, *Exserohilum* sp., *Colletotrichum* sp. and *N. sphaerica*, respectively.

### Effects of endophytic fungi fermentation on parasitism rate of *T. chinensis*

As can be seen from Table 3, daily fermentation liquid spraying was performed on the strains with high cell wall degradation enzymes, and it was found that the parasitism rates of these three strains were all improved to varying degrees, among which strain of *P. arceuthobii* had the best parasitism rate, reaching 17%, significantly higher than the control. Cell wall degrading enzymes produced by endophytic fungi contribute to the parasitism of *T. chinensis*.

**Table 3:** Effects of endophytic fungi fermentation on parasitism rate of *T. chinensis* (indoor).

Strain	Number of haustorial invasion particles/100				The parasitizing rate
	30 d	40 d	50 d	60 d	
*Pestalotiopsis* sp.	9	10	13	17	17%a
*Colletotrichum* sp.	8	9	12	16	16%a
*D. phaseolorum*	7	10	12	14	14%b
Control (water)	3	5	9	10	12%c
Liquid medium (without endophytic fungal strains)	3	7	10	11	11%c

Different lowercase letters in the same column represented significant difference (*P* < 0.05).

## Discussion

Enzymes related to cell wall degradation play an important role in the process, and endophytic fungi are one of the sources of these enzymes. Therefore, in this study, five endophytic fungi with high cell-wall degradation enzymes were screened from 147 endophytic fungi from *Taxillus chinensis* branches of different hosts. The five endophytic strains were *Colletotrichum* sp., *Nigerrospora sphaerica*, *Exserohilum* sp., *Diaporthe phaseolorum* and *Pestalotiopsis* sp., respectively. At the same time, we also isolated 47 endophytic fungi from different host branches (Supplementary Table 1). In addition to *M*orus *alba*, endophytic fungi of the other six host branches were sequenced and blasted in GenBank to *Colletotrichum* sp., *Nigerrospora* sp., *Diaporthe* sp. and *Pestalotiopsis* sp. However, further research is needed to determine whether these fungi can also produce these cell-wall degrading enzymes and whether they can promote the parasitism of *T. chinensis*. There are many kinds of cell wall degrading enzymes. In this study, four kinds of cell wall degrading enzymes were measured, including lignin peroxidase, manganese peroxidase and laccase and cellulose. We tried to use these endophytic fungi with high output of four enzymes to conduct fermentation broth culture, and selected the fermentation broth of the strain with high enzyme activity to spray *T. chinensis* seeds on branches of *M*orus *alba* every day, and calculate mulberry parasitic rate, 5% higher than the control (this part of the experiment is still repeated verification and statistics). In addition to these enzymes, analysis of previously published RNA-seq data showed that certain genes encoding xyloglucan endoglucase/hydrolases and pectin methylesterases, polygalacturonases, and cellulase-like enzymes in *Cuscuta campestris*^6,7,15,39,40^. Studies have shown that endoglycosylation of xyloglucan is most evident in the cell wall of the haustorial enlargement region towards the host plant, but can also be detected in the later stages of haustorial development^39^. These data confirm that haustorial invasion of *Cuscuta* species is facilitated not only by mechanical action but also by specific biochemical degradation and modification of host cell wall^3,40^. These researchers suggest that enzymes involved in the degradation and modification of host cell walls remodeling in *Cuscuta* XTHs prepares the parasite for host infection and may contribute to invasive haustral growth^39^. Therefore, whether these enzymes also exist in the dominant fungal isolated from different hosts and *T. chinensis*, and whether these enzymes can promote haustorium parasitism in the host, needs further experimental verification.

In this study, wheat bran was selected as the substrate of lignin fiber biomass for fermentation, and the activities of laccase, manganese peroxidase, lignin peroxidase and cellulase were determined at different times. It was found that the maximum activity time of the five strains producing laccase and cellulase was 7 days, and the highest activities of laccase and cellulase were found in strain P6, which were 117.66 and 1.66 U/mL, respectively. *Pestalotiopsis* sp. was identified by their morphological and molecular biology characteristics. It has been reported that *Pestalotiopsis* sp. fungus can produce relatively high laccase and cellulose activities, and can effectively degradation forest litter^41^. Moreover, it was found that the color of fermentation broth extracts of *Pestalotiopsis* sp. was significantly lighter than those of the other four strains. This is because laccase can be used with the decolorization and degradation of fuel and can potentially be used against global warming strategies^42,43^. *Pestalotiopsis* sp. produced the highest laccase activity of 117.66 U/mL, which was a relatively high value when compared to the unpurified crude enzyme activities reported at present, and its production did not depend on the addition of some inducible factors such as soil temperature 80 °C, ferulic acid, Cu^2+^ or dimethylaniline. Moreover, it was reported by Cao et al.^44^ that the enzyme activity of laccase with the addition of a soil temperature of 80 °C and a Cu^2+^ inducer was indeed much higher within a week of growth. However, except for the white rot fungus, *Ganoderma applanatum*, the average value of laccase activity of other two white rot fungi (*Trametes hirsuta* and *Fomes fomentarius*) did not reach their peak values. These results indicate that *Pestalotiopsis* sp. is a relatively effective laccase producing strain.

The peak activities of manganese and lignin peroxidases of the *D. phaseolorum*, *Colletotrichum* sp., *N. sphaerica* and *Exserohilum* sp. also appeared on day 7, but the second peak of manganese peroxidase activities of strain *N. sphaerica* and *Pestalotiopsis* sp. on day 11 were 8.59 and 6.79 U/mL, respectively. The enzyme activities of lignin peroxidase were 3.4 and 4.21 U/mL, respectively, which were consistent with the reports regarding lignase and cellulase of some white rot fungi^45–47^. The second peak may be due to the release of the corresponding intracellular enzymes that were originally bound to the cell membranes due to autolysis of the hyphae^41^. The four strains of N6, 4, 15 and 31 were identified by their morphological and molecular biology characteristics as *D. phaseolorum, Colletotrichum* sp.*, N. sphaerica, Exserohilum* sp., respectively. This is the first report of four fungi which produce lignocellulose-degrading enzymes using wheat gluten fibrous biomass as a substrate. Some previous studies have also investigated the activities of laccase, lignin peroxidase, manganese peroxidase and cellulase produced by fungi of six less common fungi (*Alternaria* sp., *Penicillium* sp., *Cephalosporium* sp., *Tricherderma* sp., *Pestalotiopsis* sp. and *Aspergillus fumigates*) during the degradation of Masson pine leaf litter^41^.

The degradation of lignin and cellulose is not dependent on a single enzyme, but the result of the interactions of several enzymes. Lignin degradation enzymes mainly consist of three enzymes: lignin peroxidase, manganese peroxidase and laccase^48^. Moreover, the size of the color circle is necessarily related to the level of laccase activity, but there is not a positive linear correlation between these two parameters^49^. Cellulose-degrading enzymes mainly consist of an endoglucanase, an exoglucanase and β-glucosidase, and the synergistic action of these three enzymes is required for the complete hydrolysis of cellulose into monosaccharides^50–52^.

## Materials and methods

### Sample collection of *T. chinensis*

In January 2020, the roots of *T. chinensis* from different hosts including *M. alba*, *P. salicina*, *P. chinense*, *D. odorifera*, *B. purpurea*, *D. kaki* and *D. longann* were collected in the *T. chinensis* planting base of Cenxi Funing Village, Wuzhou City (111° 51′ 14″ E, 22° 58′ 12″ N). We collected 3–5 haustoria of *T. chinensis* from the same host plants.

### The raw materials

The wheat bran was purchased from Zhonghe Modern Agricultural Development Group Co. Ltd. The pretreatment of wheat bran was based on Tao Yanjuan's method^53^ with some modifications. The wheat bran was crushed through a 40-mesh sieve, then 10 times volume of distilled water was added, and the mixture was crushed by using a colloid mill for 25 min. The liquid impurities were filtered out with a 150-mesh sieve, and the solid parts were dried in an oven at 60 °C for 24 h and then crushed. Ten times volume of distilled water was added and heated at 95 °C for 30 min. 1 M hydrochloric acid was used to adjust pH to 5.6, and 1.5% (w/w) high-temperature resistant α-amylase was added. The reaction was stirred at 95 °C for 30 min, and the complete reaction was detected with iodine solution. The temperature was lowered to 50 °C and the pH was adjusted to 9.0 with NaOH 3% (w/w) alkaline protease was added, and the reaction was stirred for 2 h, then the supernatant was discarded, filtered and rinsed through a 150-mesh sieve in clear water until the turbidity was reduced from the washed solution, and the remaining solid substances were dried in a 60 °C oven for 24 h. The wheat bran was obtained by drying and it was crushed by a micro grinder, screened with 100 mesh, dried in a constant temperature oven at 50 °C overnight and then stored. The purpose of pretreatment is to remove starch and protein from wheat bran. In this way, the main component of wheat bran dietary fiber is insoluble dietary fiber are obtained.

### Culture medium

PDA medium consisted of 200 g/L potato, 20 g/L glucose and 20 g/L agar. The pH was 7.0 and it was sterilized at 1 × 10^5^ Pa for 30 min. Seed liquid medium consisted of 20 g/L glucose, 2 g/L yeast extract, 3 g/L KH_2_PO_4_, 1.5 g/L MgSO_4_·7H_2_O and 0.5 g/L VB_1_. The pH was neutral and it was sterilized at 1 × 10^5^ Pa for 30 min. Solid medium with guaiacol consisted of 200 g/L potato, 20 g/L glucose, 20 g/L agar, 3 g/L KH_2_PO_4_, 1.5 g/L MgSO_4_·7H_2_O, 0.02 g/L VB_1_ and 1 g/L guaiacol. The pH was neutral and it was sterilized at 1 × 10^5^ Pa for 30 min. Basic medium for liquid fermentation consisted of 30 g/L wheat bran, 3 g/L KH_2_PO_4_, 1.5 g/L, MgSO_4_·7H_2_O, 1.4 g/L (NH_4_)_2_SO_4_, 0.3 g/L CaCl_2_, 5 mg/L FeSO_4_·7H_2_O, 1.6 mg/L, MnSO_4_·H_2_O and 0.02 g/L VB_1_. The pH was neutral and it was divided into 250 mL triangular flasks, or 100 mL per bottle and sterilized at 1 × 10^5^ Pa for 30 min. Cellulose solid medium^54^ was consisted of 5 g/L sodium carboxymethyl cellulose, 0.5 g/L yeast extract, 0.5 g/L peptone, 0.3 g/L, beef extract, 3 g/L, KH_2_PO_4_, 5 g/L K_2_HPO_4_, 2 g/L, (NH_4_)_2_SO_4_, 0.4 g/L MgSO_4_, 0.1 g/L CaCl_2_, 1 mL of a trace element solution, 20 g/L agar powder and 0.5 g Congo red powder dissolved in 50 mL sterile water. The trace element solution consisted of 0.07 g/L ZnCl_2_, 0.1 g/L MnCl_2_·4H_2_O, 0.06 g/L H_3_BO_3_, 0.2 g/L CoCl_2_·6H_2_O, 0.02 g/LCuCl_2_·2H_2_O, 0.02 g/L NiCl_2_·6H_2_O, 0.04 g/L NaMoO_4_·2H_2_O, and 1 ml/Lhydrochloric acid.

### Isolation and purification of endophytic fungi

Healthy and disease-free haustorium of *T. chinensis* from different hosts were selected and the tissues were cut into 5 cm fragments. These samples were washed with tap water and dried naturally. The surfaces were successively disinfected with 75% ethanol and 0.1% HgCl_2_ for 2.5 min, and washed with sterile water three times^55^. Using sterile forceps and scalpels, they were cut into tissue blocks of about 5 mm in size, and then placed on PDA (medium containing streptomycin) plates with 5 blocks per plate and 3 plates per sample. The cultures were kept at 28 °C until the mycelium grew from the edge of the tissue blocks, and then these were transferred to PDA plates for purification and preservation.

### Primary screening of ligno-cellulose degrading enzyme strains

0.1% guaiacol PDA medium was added for initial screening of lignin degrading enzymes^56^. After purification, fuangal blocks with a diameter of 6 mm were selected and placed on the plates on a super-clean platform, with 3 replicates for each strain, and these were cultured at 28 °C for 10 days. The diameters of colony and color circles within the plate were observed and statistically analyzed, as well as any changes in colony color.

Primary screening of cellulolytic enzyme strains was carried out by the following method. The tissue blocks (6 mm in diameter) of the purified strains were inoculated on the culture medium together with cellulose solid medium. For each group this was repeated 3 times and then incubated at 28 °C for 10 days. The cells were stained with 0.1% Congo red for 15 min, and then decolorized with 1 M NaCl for 15 min. Unit transparent circle diameter = transparent circle diameter − colony diameter. It was deemed that the larger the diameter of the transparent circle, the higher the enzyme production activity^54^.

The lignin cellulose-degrading enzyme strains with chromogenic and transparent rings were screened according to the above method.

### Liquid enzyme preparation

The lignocellulose-degrading strains with chromogenic and transparent rings were selected for 5–7 d cultures, and these were quantitatively inoculated respectively. 10 mL of the shaken seed liquid was added into a 250 mL triangular flask containing 100 mL of liquid fermentation medium. Each strain was inoculated in 2 bottles, and 5 strain blocks of 6 mm in diameter put the same bottle. These were incubated for 11 days in a constant temperature shaker at 26 °C and 140 r/min. Samples were taken from the fourth day of liquid culture, once daily. The fermentation liquid was filtered with four layers of gauze, and the filtrate was centrifuged at 3000 r/min for 15 min. The supernatants were the crude enzyme liquid.

### Identification of endophytic fungi

The colony morphology was recorded by referring to Fang Zhongda's method^52^, and this parameter was initially identified by referring to Wei Jingchao's method^57^. Its rDNA (ITS1 5′-TCCGTAGGTGAACCTGCGG-3′ and ITS4 5′-TCCTCCGCTTATTGATATGC-3′) and beta-tubulin (βT-2a) 5′-AACATGCGTGAGATTGTAAGT-3′ and beta-tubulin (βT-2b) 5′-ACCTCAGTGTAGTGACCTTGGC-3′) were used for molecular biological identification^58,59^. Mightyamp DNA Polymerase Ver.3 (1.25U/50μL) kit (Takara Bio Inc., Japan, Cat. No. R076A) was used to select endophytic fungal hyphae as templates for the PCR reaction. All PCR reactions were con-ducted in 50-uL volumes containing 1x PCR buffer, 0.2 mM concentrations of each dNTP, 4 mM MgCl_2_, 0.5 uM concentrations of each primer, 0.5 units of Taq DNA polymerase (Takara), and 1 uL of tem-plate DNA (20 ng/uL). The PCR program for TUB2 included a denaturation step at 94 °C for 2 min, followed by 35 cycles at 94 °C for 45 s, 60 °C for 45 s, and 72 °C for 1 min, and a final cycle at 72 °C for 10 min. The PCR program for the ITS region included a 2-min denaturing step at 94 °C followed by 34 cycles at 94 °C for 1 min, 55 °C for 30 s, and 72 °C for 1 min, and a final cycle of 10 min at 72 °C. The target bands were detected by using the gel imaging method, and the PCR products of the target bands were sent to BGI (Guangzhou) Co. Ltd. for sequencing. The sequencing results were compared using BLAST with the sequences in the NCBI GenBank. Sequence alignments of each gene and combined genes were made with ClusstalX version 1.83. The phylogenetic tree of ITS and tubulin gene was constructed using the Neighbor-Joining method with MEGA 7.0 software^60^.

### Determination of enzyme activity

The enzyme activity of laccase was measured using ABTS as substrate^61^, which was defined as the amount of enzyme required for the catalytic oxidation of 1 μmol of substrate ABTS at 30 °C per minute as a unit of laccase activity. The oxidation of substrate ABTS was determined by measuring the light absorption value of the reaction solution at 420 nm, and using a molar extinction coefficient of 36,000 mol^−1^ cm^−1^. Lignin peroxidase activity was measured by using veratrol as an oxidation substrate to produce veratrol at 30 °C^62^. A unit of enzyme activity was defined as the change of absorbance value of 0.1 unit per mL of the reaction solution per minute. The activity of manganese peroxidase was measured by the phenol red method^63^. A unit of enzyme activity was defined as the increase of the light absorption value per mL of the reaction solution at 610 nm by 0.1 unit, expressed as U mL^−1^.

Cellulase activity was determined by using a cellulase (CL) activity detection kit (Beijing Solaibuo Technology Co., Ltd.) and the 3.5-dinitrosalicylic acid (DNS) method. Under the action of cellulase, cellulose degrades to produce a reducing sugar, and the amount of reducing sugar was determined to determine enzyme activity. From the kit, 1 mL ultra-pure water was added to 10 mg anhydrous glucose standard (dry weight loss < 0.2%) to prepare a 10 mg mL^−1^ glucose solution, which was then diluted into 1.0, 0.8, 0.6, 0.4, 0.2, 0.1 and 0 mg mL^−1^ by gradient dilution. These were read at 540 nm to establish a standard curve. 1 mL of fermentation liquid was weighed and the fungi were lysed in an ultrasonic ice bath. The supernatants were centrifuged at 4 °C for 10 min at 8000 g and 2 mL of the crude enzyme liquid in centrifuge tubes were placed on ice to be measured. The reaction system was composed of 350 μL of substrate and 50 μL sample. After the reaction, the saccharification solution was obtained by boiling in order to terminate the reaction. 50 μL of the saccharification solution was taken and 150 μL DNS reagent was added and mixed. Then 1050 μL of double distilled water was added to measure the absorbance at 540 nm under the UV spectrophotometer^64,65^.

### Effects of endophytic fungi fermentation on parasitism rate of *T. chinensis*

The prepared fermentation liquid was sprayed with *T. chinensis* seeds parasitized on *Morus alba* every day until 20 d, and water and without the addition of endophytic fungal strains liquid were used as control. The parasitizing rate was calculated every 10 d until 60 d, and the final parasitizing rate was calculated. The parasitizing rate was based on haustorium entering the host completely. The fermentation liquid is the same as above (Liquid Enzyme Preparation).

### Statistical analysis of data

Statistical results were expressed as x ± s, with ± being the mean value and s being the standard deviation. SPSS19.0 software was used to conduct univariate ANOVA analysis and the variance homogeneity test for data in each group. *P* < 0.05 was considered statistically significant.

## Supplementary Information

Supplementary Information 1.

Supplementary Information 2.

Supplementary Information 3.
