# Supplementary figures and images for: Effects of endophytic fungi on parasitic process of Taxillus chinensis
Source: Sci Rep. 2022 May 11;12:7744. doi: 10.1038/s41598-022-11940-z (PMC9095678; doi:10.1038/s41598-022-11940-z)

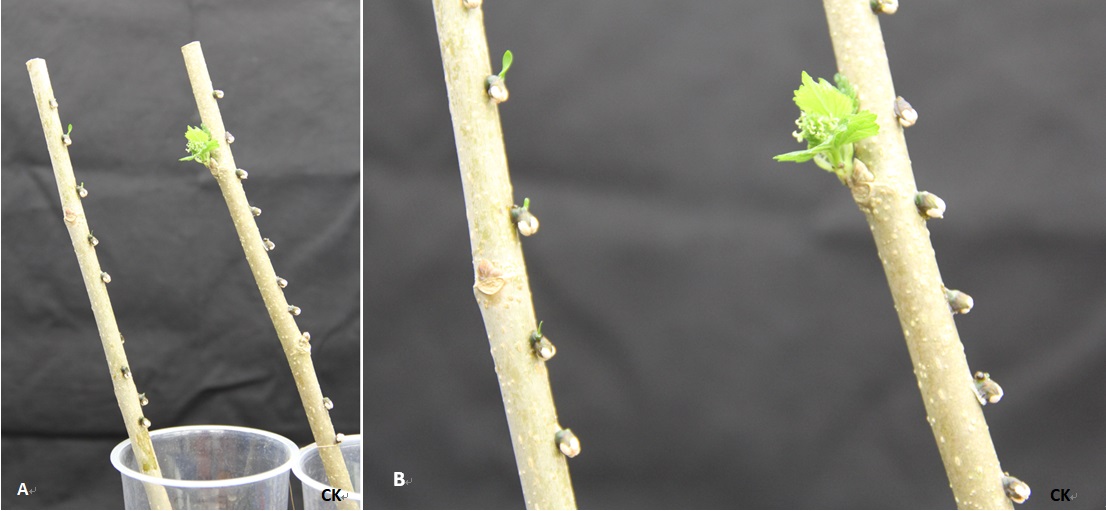

Supplement: Supplementary file 1 — Supplementary Information 1. [file 41598_2022_11940_MOESM1_ESM.jpg]

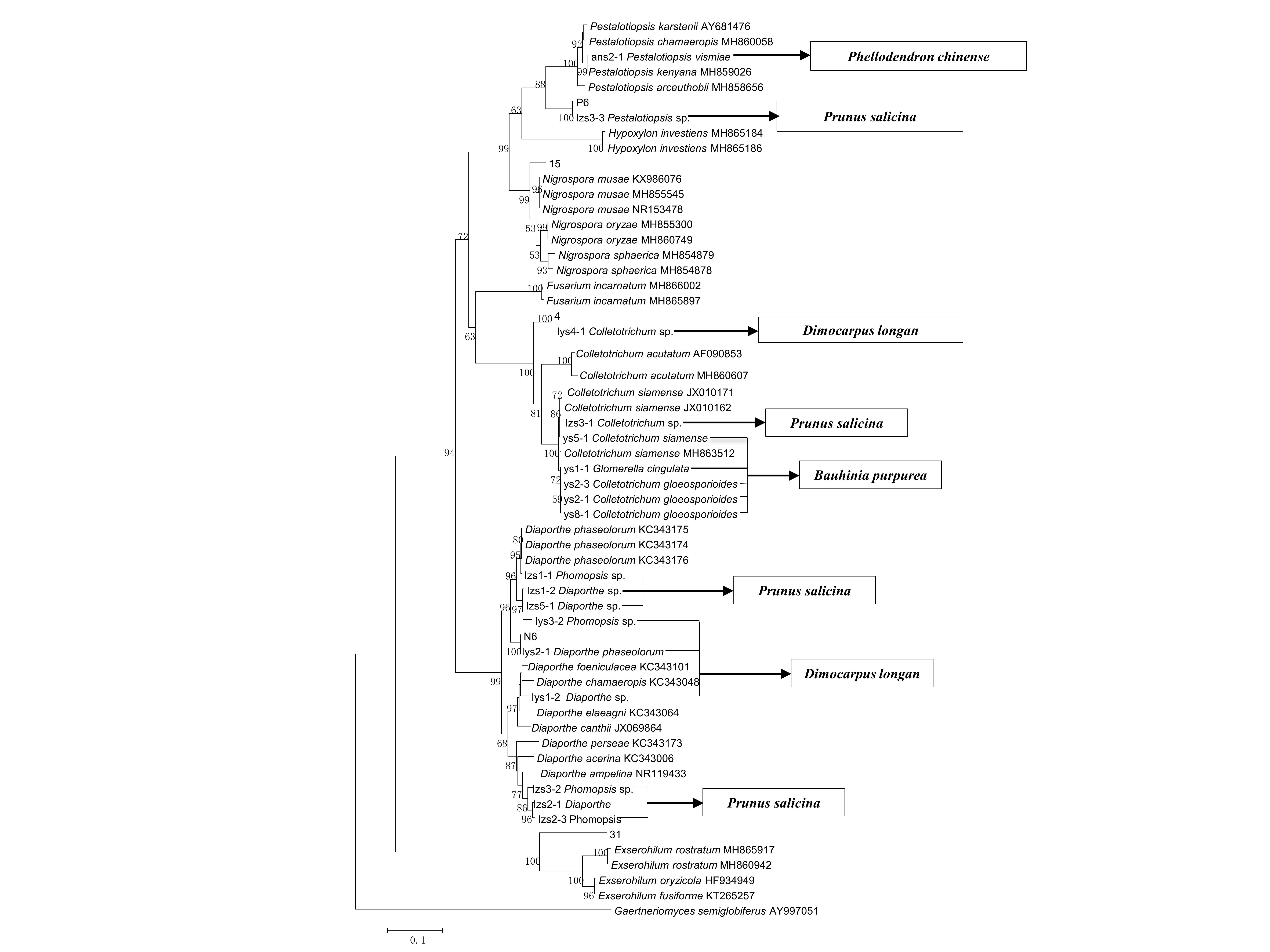

Supplement: Supplementary file 2 — Supplementary Information 2. [file 41598_2022_11940_MOESM2_ESM.jpg]
